# Molecular Detection of *Anaplasma marginale* in *Amblyomma mixtum* Infesting Cattle in the Major Livestock-Producing States of Mexico

**DOI:** 10.3390/pathogens14030214

**Published:** 2025-02-21

**Authors:** Carolina Cárdenas-Amaya, Dora Romero-Salas, Marta Rafael, Jenny J. Chaparro-Gutiérrez, Sara López-Osorio, Mariel Aguilar-Domínguez, Miguel Á. Alonso-Díaz, Adalberto Á. Pérez de León, José de la Fuente

**Affiliations:** 1 Laboratorio de Parasitología, Rancho “Torreón del Molino”, Facultad de Medicina Veterinaria y Zootecnia, Universidad Veracruzana, Carretera Veracruz-Xalapa Km. 14.5, Col. Valente Díaz, Veracruz 91697, CP, Mexico; carolina930527@gmail.com (C.C.-A.); marieaguilar@uv.mx (M.A.-D.); 2 Grupo de Investigación en Sanidad y Biotecnología (SaBio), Instituto de Investigación en Recursos Cinegéticos (IREC), Consejo Superior de Investigaciones Científicas (CSIC), Universidad de Castilla-La Mancha (UCLM)-Junta de Comunidades de Castilla-La Mancha (JCCM), Ronda de Toledo 12, 13005 Ciudad Real, Spain; marta.srafael@uclm.es (M.R.); josedejesus.fuente@uclm.es (J.d.l.F.); 3 Grupo de Investigación Centro de Investigaciones Básicas y Aplicadas en Veterinaria (CIBAV), Facultad de Ciencias Agrarias, Universidad de Antioquia (UdeA), Carrera 75 No 65-87 Bloque 47-241, Medellín 050034, Colombia; jenny.chaparro@udea.edu.co (J.J.C.-G.); sara.lopezo@udea.edu.co (S.L.-O.); 4 Centro de Enseñanza, Investigación y Extensión en Ganadería Tropical (CEIEGT), Facultad de Medicina Veterinaria y Zootecnia, Universidad Nacional Autónoma de México, Km. 5.5 Carr. Fed. Martínez de la Torre-Tlapacoyan, Veracruz 93650, CP, Mexico; alonsodm@unam.mx; 5 United States Department of Agriculture-Agricultural Research Service, San Joaquin Valley Agricultural Sciences Center, 9611 South Riverbend Avenue, Parlier, CA 93648, USA; beto.perezdeleon@usda.gov; 6 Department of Veterinary Pathobiology, Center for Veterinary Health Sciences, Oklahoma State University, Stillwater, OK 74078, USA

**Keywords:** hemoparasite, anaplasmosis, three-host tick, bovine, *Anaplasma marginale*, *Amblyomma mixtum*

## Abstract

Bovine anaplasmosis is a tick-borne disease caused by *Anaplasma marginale*, although mechanical transmission by biting flies also occurs. Infection with *A. marginale* can reach 26% mortality and morbidity is associated with reduced beef and milk production, causing economic losses for livestock producers. Between March 2022 and July 2023, 1920 ticks were collected from 52 cattle production units in major cattle-producing states in Mexico, including Chiapas, Jalisco, Michoacán, Tabasco, and Veracruz. Of all the ticks collected, 35.57% were morphologically identified as *Amblyomma mixtum*. Samples of *A. mixtum* from each state, totaling 271, were tested for *A. marginale* via polymerase chain reaction (PCR). *A. marginale* was detected molecularly in 15.3% of *A. mixtum* samples. *A. mixtum* from Chiapas had the highest prevalence of *A. marginale* (24.0%), followed by Tabasco and Veracruz (20.0% each), Jalisco (15.2%), and Michoacán (6.6%). Phylogenetic analysis supported the morphological identification of *A. mixtum* and confirmed the genetic identity of *A. marginale*. This research is the first report on the molecular detection of *A. marginale* in *A. mixtum* ticks in Mexico. Results suggest that this 3-host tick species might be a potential vector. *A. mixtum* is Mexico’s second most economically significant tick feeding on cattle after *Rhipicephalus microplus*. This information about *A. marginale* prevalence in *A. mixtum* expands our understanding of bovine anaplasmosis epidemiology in Mexico. Further research is needed to assess the role of *A. mixtum* as a vector of *A. marginale*.

## 1. Introduction

*Anaplasma marginale* is a tick-borne intracellular pathogen of the Anaplasmataceae family (order Rickettsiales) [1] and the causative agent of bovine anaplasmosis, which is of great economic importance for the livestock industry globally [2,3]. Bovine anaplasmosis causes severe hemolytic anemia, affecting the health and productivity of infected cattle, leading to decreased milk and meat production and, in severe cases, mortality [2,4]. Production losses, treatment costs, and associated control measures make bovine anaplasmosis one of the most economically impactful tick-borne infections globally [5,6]. In endemic areas, the economic impact is potentially severe due to the increased costs incurred during attempts to manage the disease at the herd level while losses are registered in terms of reproduction and beef production [7,8].

In addition to natural tick vectors, *A. marginale* can be transmitted to cattle by biting flies and iatrogenic means [1]. Bovine anaplasmosis is challenging to control due to *A. marginale’s* ability to establish persistent infections in its hosts, including via cattle and tick vectors, allowing continuous transmission within affected herds [9,10]. This persistence is mediated by molecular mechanisms that enable the pathogen to evade the host’s immune system, further complicating its management [11,12].

The global distribution of *A. marginale* includes tropical and subtropical regions in the Americas, Africa, and Asia, with multiple genotypes that vary in terms of infectivity and transmission capacity across different tick species [1,13]. The genetic diversity of *A. marginale* contributes to regional differences in disease severity and the success of control strategies in various geographic areas [7,14]. Bovine anaplasmosis is endemic in Mexico, where infection with *A. marginale* is reported in cattle and other species, like water buffalo, that may play a significant epidemiological role as reservoirs [15,16].

*Rhipicephalus microplus* and *R. annulatus* are known tick vectors of *A. marginale* in Mexico [16]. Anecdotal evidence suggests that the three-host tick, *Amblyomma mixtum*, may also serve as a vector of *A. marginale* in Mexico, where this tick-borne pathogen has a documented prevalence of 70% in cattle [17]. *A. mixtum* is regarded as the second most economically important tick affecting livestock in Mexico after *R*. *microplus* and can co-infest cattle alongside this one-host tick [18,19]. However, the role of *A. mixtum* in the transmission of *A. marginale* and its involvement in the epidemiology of bovine anaplasmosis remain to be determined.

*A. mixtum* is part of the *A. cajennense* species complex, with a wide distribution in Mexico and other Latin American countries [19,20,21]. Zoonotic pathogens including *Rickettsia* spp. and *Ehrlichia* spp. are known to be transmitted by *A. mixtum* [22]. Given its role as a vector of other pathogens, the ability of *A. mixtum* to transmit *A. marginale* may depend on environmental factors as well as tick population density in livestock areas [14,23].

Implementing molecular detection methods to monitor the presence of tick-borne pathogens like *A. marginale* in tick populations could enhance our epidemiological understanding and improve the control of bovine anaplasmosis [24]. This study aimed to detect the presence of *A. marginale* in *A. mixtum* and ascertain its prevalence in Mexico’s main livestock-producing states. The molecular detection of *A. marginale* in *A. mixtum* reported herein underscores the need to adapt suitable management plans and control measures to prevent cases of bovine anaplasmosis in areas of Mexico where *A. mixtum* infests cattle.

## 2. Materials and Methods

### 2.1. Study Area

**Figure 1:**
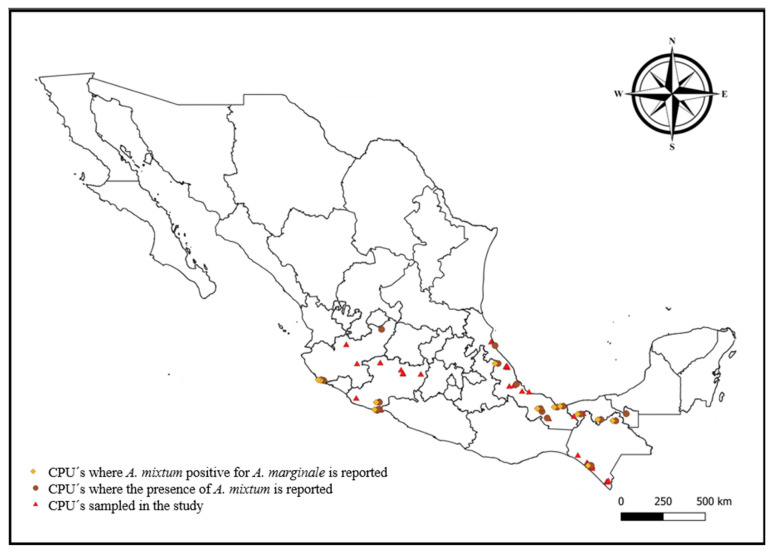
A geographical representation of CPUs sampled by state in Mexico, including CPUs reporting the presence of *Amblyomma mixtum* and the molecular detection of *Anaplasma marginale* in this tick. The map was created using QGIS (Geographic Information System).

A descriptive study was conducted, using a convenience sampling method, in order to collect ticks from cattle belonging to 52 cattle production units (CPUs) located in the primary Mexican states with the largest cattle inventories, including Chiapas, Jalisco, Michoacán, Tabasco, and Veracruz [25] (Table 1). Tick sampling took place from March 2022 to July 2023. The location of sampling sites within each state was determined based on published data describing the potential distribution of *A. mixtum* in these regions [21] (Figure 1). Most CPUs comprised other animals, including horses, dogs, laying hens, and pigs.

### 2.2. Collection and Taxonomic Identification of Specimens

Each bovine was inspected from head to tail and against the hair grain to locate ticks, as described [19]. Ticks were removed using entomological forceps by applying gentle vertical traction [26]. Each specimen was individually stored in vials containing 70% ethanol (*v*/*v*) for preservation. Tick identification was performed using a MOTIC^®^ (Microscope World, Carlsbad, CA, USA) stereoscopic microscope and dichotomous taxonomic keys. Different taxonomic keys were used to distinguish *A. cajennense* [27], *A. mixtum* [20], and *R. microplus* [28].

### 2.3. DNA Extraction

Specimens identified as *A. mixtum* were individually processed using the TRI reagent (Sigma-Aldrich, St. Louis, MO, USA), a mixture of guanidinium thiocyanate and phenol, following the manufacturer’s protocol. DNA concentration and purity were assessed using a Nanodrop ONE^®^ spectrophotometer (Thermo Fisher Scientific, Waltham, MA, USA) via the quantification of nucleic acids at an optical density of 260 nm, with the ratio of absorbance set at 260/280 nm.

### 2.4. Polymerase Chain Reaction (PCR) Test

The quality of the DNA extraction protocol and tick species confirmation were appraised through the conventional polymerase chain reaction (PCR) of the 16S ribosomal DNA (16S rDNA) [29] gene and the cytochrome oxidase subunit I (COI) gene of 26 individual ticks [30] (Table 2). For pathogen detection, specific oligonucleotides and conditions were used to amplify a fragment of the 16S rDNA gene for *Anaplasma* spp. Subsequently, a second PCR procedure was performed to amplify the msp5 region corresponding to *A. marginale* using the conditions and oligos described previously [31] (Table 2).

PCR amplifications were performed in a total reaction volume of 25 µL, containing 12.5 µL of 2× PCR Master Mix (Thermo Fisher Scientific), 0.5 µM of each primer, 2 µL of DNA template (~50 ng), and nuclease-free water, to complete the final volume. The PCR cycling conditions were as follows. We used 16S rDNA for tick molecular identification: we performed initial denaturation at 95 °C for 3 min, followed by 35 cycles of 95 °C for 30 s, 48 °C for 30 s, and 72 °C for 45 s, with a final extension at 72 °C for 5 min. We used COI for tick molecular identification: we performed initial denaturation at 94 °C for 3 min, followed by 35 cycles of 94 °C for 30 s, 50 °C for 30 s, and 72 °C for 1 min, with a final extension at 72 °C for 5 min. We used 16S rDNA for *Anaplasma* spp. molecular detection: we performed initial denaturation at 95 °C for 5 min, followed by 35 cycles of 95 °C for 30 s, 42 °C for 30 s, and 72 °C for 1 min, with a final extension at 72 °C for 10 min. We used msp5 for *A. marginale* molecular identification: we performed initial denaturation at 95 °C for 5 min, followed by 35 cycles of 95 °C for 30 s, 54 °C for 30 s, and 72 °C for 1 min, with a final extension at 72 °C for 10 min. Positive and negative controls were included in all PCR reactions to ensure the accuracy and reliability of the results. The positive control consisted of DNA from a confirmed *Anaplasma marginale* sample obtained from a bovine blood sample (for *Anaplasma* spp. molecular detection we used 16S DNA corresponding to *A. ovis*). The negative control consisted of nuclease-free water in order to confirm the absence of contamination. PCRs were conducted using a C1000 touch PCR thermal cycler (Bio-Rad, Hercules, CA, USA), with PCR fragments visualized in 1.5% agarose gel stained with GelRed (Biotium, Fremont, CA, USA) under UV transillumination conditions. Presumed positive samples were purified using the MinElute PCR Purification Kit (Qiagen, Hilden, Germany) and sent to Secugen in Madrid, Spain, for sequencing.

### 2.5. Phylogenetic Reconstruction

Chromas software v.2.6.6 was used to edit the sequences obtained. Homology analysis was performed using the National Center for Biotechnology Information (NCBI) database with the Basic Local Alignment Search Tool (BLAST 2.16.0). The sequences for *Anaplasma marginale* was deposited in the GenBank database (accession numbers P PV052636.1, PV052637.1 and PV052638.1). Phylogenetic reconstruction was performed using the Maximum Likelihood method. We selected the best nucleotide substitution model in the MEGA v.11 software [32] and applied the ClustalW algorithm for multiple sequence alignment [33]. We performed 1000 bootstrap replicates to assess the robustness of the phylogenetic groupings.

### 2.6. Statistical Analysis

The similarity of tick/pathogen/state variables was analyzed by applying a χ2 test and descriptive statistical analysis was performed using STATA software, version 14.0 [34]. Maps were created using QGIS (Geographic Information System) (3.38.2 version) [35].

## 3. Results

**Figure 2:**
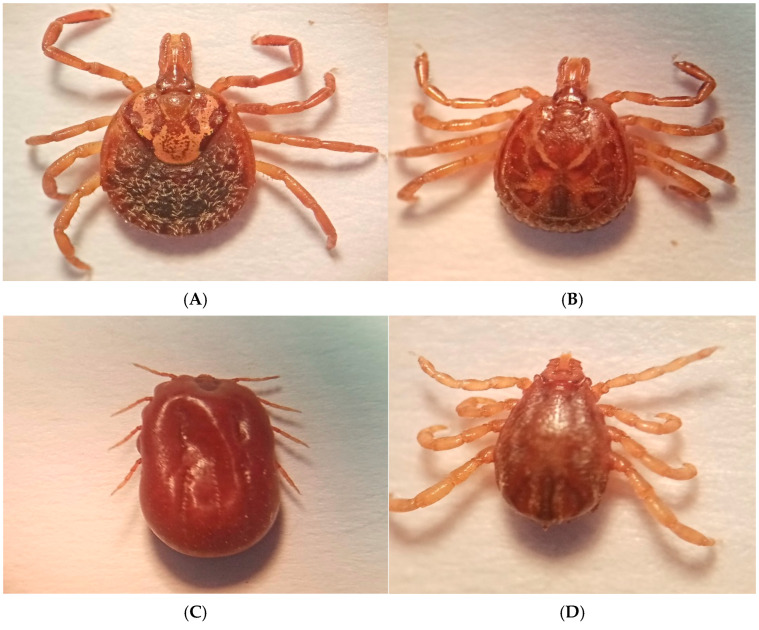
The taxonomic identity of adult ticks collected from cattle in cattle production units across the states sampled in Mexico: (**A**) *Amblyomma mixtum* (female); (**B**) *Amblyomma mixtum* (male); (**C**) *Rhipicephalus microplus* (female, engorged); (**D**) *Rhipicephalus microplus* (male).

**Figure 3:**
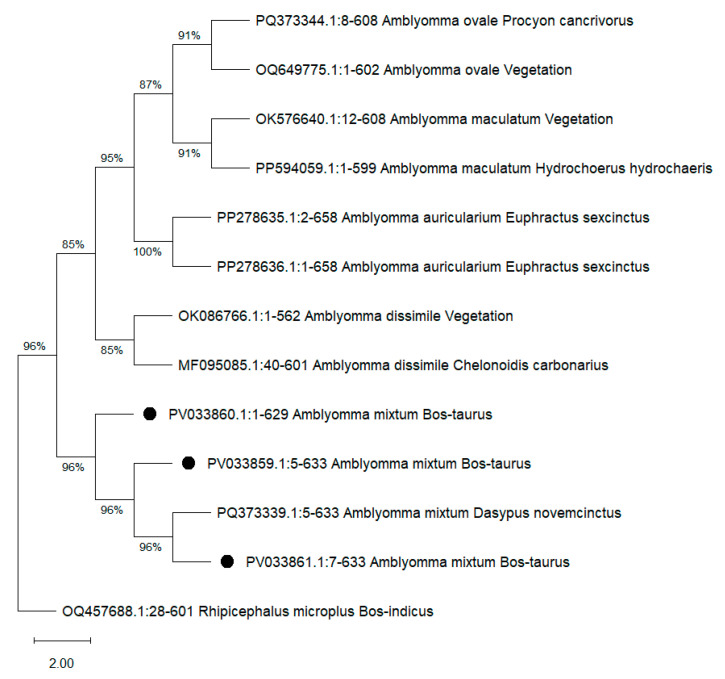
A phylogenetic tree of the partial COX1 sequence of *Amblyomma mixtum*, as inferred using the Maximum Likelihood method. Sequences from the samples in this study are marked with a black dot. GenBank accession numbers for the partial COX1 sequences are followed by the corresponding species name and the host from which they were obtained.

A total of 1920 adult ticks were collected from infested cattle. Of all the ticks collected, 64.4% were morphologically identified as *R. microplus* and 35.6% were morphologically identified as *A. mixtum* (Figure 2, Table 3). BLAST analysis of the sequences obtained for the 26 ticks tested revealed 98.9–100% identity with *A. mixtum* [GenBank accession numbers for 16S rDNA PV034243, PV034244 and PV034245, and for COX1 PV033859, PV033860 and PV033861 (Figure 3)].

PCR results revealed that all samples positive for the *A*. *marginale* 16S gene were also positive for the msp5 gene. Similarly, the samples were positive for both genes in their entirety; this was the case for species confirmation (Table 3).

A total of 271 *A. mixtum* ticks were processed for the molecular detection of *A. marginale*. For the first time, our study was able to detect the presence of *A. marginale* in *A. mixtum* (n = 47) in Mexico. The result represents an overall frequency of 17.3% (Table 3). PCR results revealed that all samples positive for the *A. marginale* 16S gene were also positive for the msp5 gene. The highest prevalence of *A. marginale* was observed in Chiapas (24.0%), followed by Tabasco and Veracruz (both at 20.0%), while Jalisco showed a prevalence of 15.2%, and Michoacán had the lowest prevalence (6.6%). Although lacking statistical significance (χ2 = 5.26, d.f. = 4, P = 0.262), these results suggest a heterogeneous geographic distribution of *A. marginale* infection in *A. mixtum* infesting cattle in the major livestock-producing states of Mexico (Table 3, Figure 1).

These results demonstrate the heterogeneous geographic distribution of A. marginale infection in A. mixtum; however, they are without representative statistical difference (χ2 = 5.26, d.f. = 4, P = 0.262) (Table 3, Figure 1).

**Figure 4:**
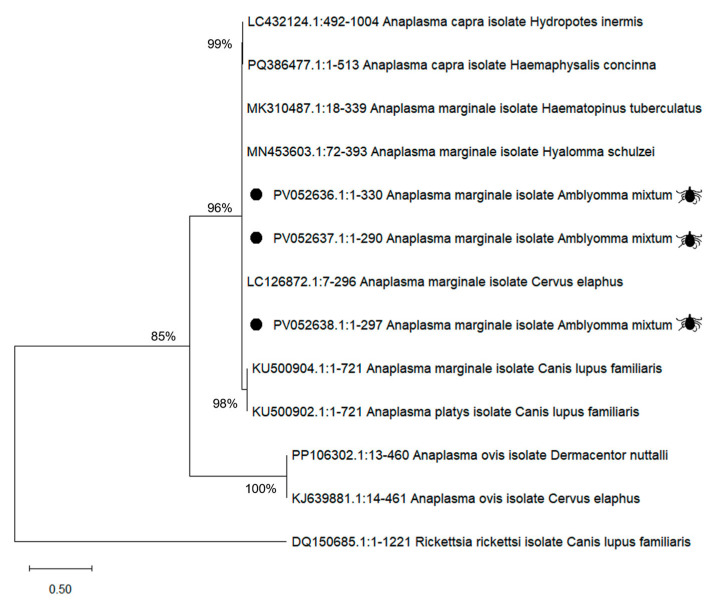
A phylogenetic tree of msp5 sequences of *Anaplasma marginale* isolated from *Amblyomma mixtum* ticks collected from Mexico. The sequences obtained in this study are marked with a tick symbol. The analysis was performed based on Maximum Likelihood Analysis with a Tamura-3-parameter model. The reliability of internal branches was assessed using the bootstrapping method with 1000 replicates.

With respect to pathogen molecular analysis, sequences of *A. marginale* msp5 obtained in this study showed high genetic identity with sequences previously reported in GenBank (Figure 4). The phylogenetic tree shows the evolutionary position of an isolate of A. marginale obtained from the tick *A. mixtum* (labeled as *Anaplasma marginale* isolate *Amblyomma mixtum*, with accession numbers PV052636.1, PV052637.1 and PV052638.1). These isolates fall within the main *A. marginale* clade, being grouped with other *A. marginale* isolates from *Cervus elephanus* (LC126872.1), as well as with other ectoparasite species such as *Hyalomma schulzei* (MN453603.1) and *Haematopinus tubercuatus* (MK310487.1).

## 4. Discussion

Our results detected, for the first time, the presence of *A. marginale* in *A. mixtum* ticks in Mexico. *A. mixtum* exposure to *A. marginale* might have occurred through co-feeding with infected *R. microplus* or by feeding on a bacteremic bovine. In this case, *A. mixtum* could be a mechanical vector, as has been shown for some biting flies where exposure to *A. marginale* results in a non-productive infection [16]. However, the vector competence of *A. mixtum* for *Rickettsia* spp. and *Ehrlichia* spp. suggests that this 3-host tick could be infected with *A. marginale* under certain conditions [22,36]. This possibility highlights the necessity for research to ascertain if *A. mixtum* can be involved in the transmission of *A. marginale* to bovine hosts.

Results from this study underscore the potential of *A. mixtum* as a vector of *A. marginale*. These findings are epidemiologically relevant for bovine anaplasmosis given the documented coexistence of *A. mixtum* and *R. microplus*, which is the principal vector of *A. marginale* in Mexico, on infested cattle. The frequency of *A. marginale* detected in *A. mixtum* showed statistically insignificant but biologically notable geographic variation among the Mexican states sampled. These differences may be influenced by environmental factors such as the specific climatic conditions of each region. For example, Chiapas and Tabasco, with tropical environments characterized by high temperatures and relative humidities [37,38], provide suitable habitats for the proliferation of *A. mixtum*, which can result in enhanced *A. marginale* transmission [14,39]. By contrast, conditions in Michoacán with relatively cooler conditions and apparently stricter livestock management practices [40,41] may be less conducive to finding cattle infested with the neotropical *A. mixtum* that are infected with *A. marginale*.

The overall detection rate obtained in this study, standing at 15.3%, agrees with previous molecular studies conducted in Latin America, where detection rates for *A. marginale* in *A. mixtum* ranged from 12% to 40% [8,24]. Factors influencing the molecular detection of *A. marginale* in *A. mixtum* included environmental conditions, vector density, and livestock management practices. Similarly, temperature, humidity, and cattle density influence the epidemiology of tick-borne diseases affecting cattle in tropical regions of South America [14,24]. However, this contrasts with the findings of [17], where *A. marginale* was not detected in *A. mixtum*. Several factors could account for these differences. First, regional variations in tick and host populations might influence the prevalence of *A. marginale*, as environmental and ecological factors can affect both the distribution of the vector and the pathogen [19]. Second, differences in sampling strategies, including the number and type of hosts from which ticks are collected, can impact detection rates. Third, methodological discrepancies, such as variations in DNA extraction protocols, target genes, or PCR sensitivity, can lead to divergent results. Lastly, temporal differences in studies may reflect changes in the epidemiology of *A. marginale* due to shifts in livestock management practices, vector control measures, or pathogen evolution over time. These factors highlight the importance of considering regional and methodological contexts when comparing findings across studies. Cattle management practices that influence the dynamics of *A. marginale* transmission include acaricide use and other tick-borne disease control measures [7]. The higher frequencies observed in tropical states like Chiapas and Tabasco also suggest that environmental conditions, combined with less stringent livestock management practices, may contribute to the high density of infected *A. mixtum* [14]. Conversely, states such as Michoacán and Jalisco, with lower prevalences, may benefit from more effective livestock management strategies or reduced contact between cattle and *A. mixtum*. Additionally, factors such as vegetation, water access, and cattle movement could also influence the geographic variation observed [42].

In Mexico, detecting and confirming the presence of *A. marginale* through molecular analysis emphasizes the need to manage populations of *A. mixtum* infesting cattle because of their potential in the epidemiology of bovine anaplasmosis [13]. The diversity of hosts *A. mixtum* parasitizes to complete its 3-host life cycle raises the specter of co-infection with pathogens that may include *A. marginale*. This multi-host life cycle complicates efforts to control vector-borne cattle diseases in areas of Mexico where *A. mixtum* and *R. microplus* coexist [23,43]. This situation reinforces the importance of an integrated approach to managing tick-borne diseases [44].

Integrated bovine anaplasmosis management would not only include interventions to control tick vectors but also measures to prevent infection and cure subclinical and clinical infections in affected bovines, taking into consideration aspects of the One Health concept [45,46,47]. Future research should evaluate the vectorial capacity of *A. mixtum* and its interaction with *R. microplus* and explore the impacts of coinfections on *A. marginale* transmission dynamics. This knowledge will allow informed decision-making to improve the prevention and management of bovine anaplasmosis in parts of the world like Mexico, where multiple tick vector species contribute to the intensity of endemic disease pressure on cattle herds [48].

## 5. Conclusions

The findings of this study support the hypothesis that *A. mixtum* is a potential vector of *A. marginale*. Molecular detection rates for *A. marginale* in *A. mixtum* collected from cattle in the main livestock-producing states of Mexico showed a heterogeneous geographic distribution. However, these differences were not statistically significant, indicating that additional factors may influence distribution and that larger studies are required to confirm this apparent trend. The highest detection rates were observed in the states of Chiapas and Tabasco, which are located in tropical areas.

A holistic approach through integrated bovine anaplasmosis management would address regional differences in tick vector ecology and disease epidemiology. Research is needed to determine if *A. mixtum* is a competent vector of *A. marginale*, its interaction with other vectors including *R. microplus*, and its impact on the risk of *A. marginale* transmission among cattle herds.

## Figures and Tables

**Table 1:** The geographical description of the cattle production units, which were sampled according to the corresponding locality, municipality, and state in Mexico.

State	Municipality	Locality	Geolocation		Elevation (m.a.s.l.)
			Latitude	Longitude	
Chiapas	Tapachula	Oro Verde	14.84200	−92.34953	10
		Corlai	14.87378	−92.36289	15
		Tapachula	14.90611	−92.29503	20
	Mapastepec	Adolfo López Mateos	15.44686	−92.99903	500
		Dos Pasajes	15.48253	−93.06236	520
		La Trinidad	15.54211	−92.99906	530
	Pijijiapan	Puente Margaritas	15.59361	−93.04472	540
		La Herradura	15.59669	−93.07642	550
		Gabriel Toledo	15.58256	−93.17053	560
		Las Carmelitas	15.60114	−93.17806	570
		Caña Brava	15.64281	−93.17767	580
		Pijijiapan	15.70444	−93.22747	600
	Tonalá	Agua Prieta	16.01653	−93.62961	700
	Palenque	Palenque	17.53831	−91.97250	100
Jalisco	Cihuatlán	Emiliano Zapata	19.29353	−104.71400	1500
	La Huerta	El Progreso	19.32539	−104.81994	1520
	Concepción de Buenos Aires	Concepción de Buenos Aires	19.99872	−103.26397	1530
	Encarnación de Díaz	Concepción de Buenos Aires	20.01097	−103.25503	1540
		La Cuadra	21.52769	−102.19153	1550
Michoacán	Lázaro Cárdenas	Lázaro Cárdenas	17.98364	−102.23978	50
		Buenos Aires	18.03692	−102.27950	55
		Playa Azul	17.98908	−102.38244	60
		El Habillal	18.02278	−102.36128	65
		El Habillal	17.99297	−102.38158	70
	Arteaga	Arteaga	18.34197	−102.29094	75
		Arteaga	18.35228	−102.28775	80
	Aquila	El Aguacate	18.49844	−103.29672	85
	Tuxpan	El Malacate	19.54581	−100.47789	1600
	Morelia	Cañada de Buena Vista	19.55433	−101.25333	1610
		Santiago Undameo	19.59053	−101.25778	1620
		Santiago Undameo	19.59289	−101.25850	1630
	Zamora	Romeo de Guzmán	20.04158	−102.24872	1640
	Cuto de la Esperanza	Cuto de la Esperanza	19.72861	−101.34217	1650
Tabasco	Balancán	El Tornillo	17.84378	−91.51683	200
	Tacotalpa	Puente de Piedra	17.59256	−92.61969	210
	Huimanguillo	Ocuapan	17.83039	−93.50228	220
Veracruz	Cosoleacaque	Calzadas	18.13028	−94.52667	230
	San Juan Evangelista	Rancho Azteca	17.65839	−94.96669	240
	Juan Rodríguez Clara	Perseveranza	17.93864	−95.19114	250
	Coatzacoalcos	Matilla de Conejo	18.06981	−95.25825	260
	Isla	Matilla de Conejo	18.06369	−95.27286	270
	Túxpam de Rodríguez Cano	Matilla de Conejo	18.17344	−94.26556	280
	Manlio Fabio Altamirano	El Tigre	17.93589	−95.36167	290
	Tlapacoyan	Guillermo Prieto	18.17344	−94.26556	280
		San Isidro	18.08114	−95.53406	300
		Lindavista	20.81628	−97.23894	310
		Mata Loma	19.13728	−96.30019	320
		San Francisco	20.03556	−97.10631	330

**Table 2:** Oligonucleotides and references for the amplified genes.

Gene	Sequence 5′-3′ (F: Forward/R: Reverse)	Fragment (bp)	Annealing (°C)	Reference
16Sr DNA	F: CCGGTCTGAACTCAGATCAAGT R:CTGCTCAATGATTTTTTAAATTGCTGTGG	460	48	[29]
COI	F: GGTCAACAAATCATAAAGATATTGG R: TAAACTTCAGGGTGACCAAAAATCA	650	50	[30]
16S rDNA (*Anaplasma* spp.)	F: CAGAGTTTGATCCTGGCTCAGAACG R: GAGTTTGCCGGGACTTCTTCTGTA	421	42	[31]
MSP5	F:GCATAGCCTCCGCGTCTTTC R: TCCTCGCCTTGGCCCTCAGA	456	54	[31]

**Table 3:** Tick distribution and prevalence of *Anaplasma marginale* in adult *Amblyomma mixtum* ticks collected from cattle in cattle production units across major livestock-producing states in Mexico.

			Tick Taxonomic Identification				*A. marginale* in *A. mixtum*			
State	CPUs Sampled	Total Ectoparasites	*R. microplus*	(%)	*A. mixtum*	(%)	*A. mixtum* Processed	Positive for *A. marginale*	(%)	IC 95%
Chiapas	14	196	161	82.2	35	17.8	25	6	24	11.5–43.4
Jalisco	8	260	214	82.3	46	17.7	46	7	15.2	7.5–28.2
Michoacán	14	364	319	87.6	45	12.4	45	3	6.6	22.9–17.8
Tabasco	4	144	66	45.8	78	54.2	75	15	20	12.5–30.4
Veracruz	12	956	477	49.9	479	50.1	80	16	20	12.7–30.0
Total	52	1920	1237	64.4	683	35.6	271	47	17.3	13.3–22.3

## Data Availability

All sequences obtained were deposited in the GenBank database, whose accession numbers were mentioned above, in the following link the accession number is placed and complementary information is provided: https://blast.ncbi.nlm.nih.gov/blast/Blast.cgi?PROGRAM=blastn&PAGE_TYPE=BlastSearch&LINK_LOC=blasthome (accessed on 27 December 2024).
